# Therapeutic efficiency of rhenium-188-HEDP in human prostate cancer skeletal metastases

**DOI:** 10.1038/sj.bjc.6601158

**Published:** 2003-08-12

**Authors:** K Liepe, J Kropp, R Runge, J Kotzerke

**Affiliations:** 1 Department of Nuclear Medicine, University Hospital Dresden, Fetscherstr. 74, 01307 Dresden, Germany

**Keywords:** rhenium-188-HEDP, prostate cancer, bone metastases, therapeutic efficiency

## Abstract

Rhenium-188-HEDP (^188^Re-HEDP) is a new and attractive radiopharmaceutical for the treatment of metastatic bone pain. As a product of ^188^W/^188^Re generator, it is convenient for clinical therapeutic use with a short physical half-life of 16.9 h and a maximal *β*-energy of 2.1 MeV. We investigated the effect of ^188^Re-HEDP on pain relief, analgesic intake and impairment of bone marrow function in 27 patients with bone metastases induced from prostate cancer. All patients were interviewed using a standardised set of questions before, and after therapy for 12 weeks. The patients were treated with 2700–3459 MBq of ^188^Re-HEDP. Blood samples were taken weekly for 12 weeks, and a blood count was performed. Patients described an improvement on the Karnofsky performance scale from 74±7 to 85±9% 12 weeks after therapy (*P*=0.001). The pain score showed a maximum decrease from 44±18 to 27±20% in the third to the eight week after therapy (*P*=0.009). Seventy-six percent of the patients described a pain relief without increase of analgesic intake. Twenty percent of the patients could discontinue their analgesics and were pain free. Mean platelet count decreased from (286±75)*10^3^ *μ*l^−1^ to (215±92)*10^3^ *μ*l^−1^, and mean leucocyte count from (7.7±1.5)*10^3^ *μ*l^−1^ to (6.0±1.9)*10^3^ *μ*l^−1^ in the second to the fourth week after therapy. The maximal differences between the values of platelets and leucocytes before and after therapy were not statistically significant (*P*=0.021 and 0.094). In conclusion, ^188^Re-HEDP is an effective radiopharmaceutical used in the palliative treatment of metastatic bone pain in prostate cancer and shows minimal bone marrow toxicity.

Prostate cancer is the second common cause of cancer mortality in men in the UK and in the US (American Cancer Society, 1991; Office for National Statistics Series, 1996). Prostate cancer accounts for 17% of malignant diseases in men >50 years in Germany (‘Krebsregister in Deutschland’, 1999) with a 5-year survival rate of 70%.

The skeleton is the most common site for prostatic metastases. The major mechanism of pain from small metastases appears to be the stimulation of nerve endings in the endosteum by a variety of chemical mediators. Larger bone metastases produce stretching of the periosteum that leads to pain (Nielsen *et al*, 1991). Resulting bone pain interferes with the patient's quality of life and requires effective treatment. Unfortunately, various nonradiotherapeutic modalities such as analgesics, hormone therapy, orchidectomy, cytostatic and cytotoxic drugs, bisphosphonates and surgery are not effective in all cases, especially in the late stage of the disease (McEwan, 1998; Debes and Tindall 2002; Gilligan and Kantoff, 2002). External-beam radiotherapy is suitable only for well-defined localised bone metastases. Extended field radiation may be useful in patients with diffuse metastases, but is often accompanied by serious side effects (Lewington, 1993). Therefore, systemic radionuclide therapy must be considered as a valuable and effective method of treatment in patients with widespread skeletal metastases.

The various radiopharmaceuticals that are used for palliative treatment of bone metastases include strontium-89 (Pecher, 1942Pl. check Pecher or Pecher et al) phosphorus-32 (Friedell and Storaassli, 1950), iodine-131-BDP3 (Eisenhut *et al*, 1986), yttrium-90 (Kutzner *et al*, 1991), rhenium-186-HEDP (Maxon *et al*, 1990) and samarium-153-EDTMP (Turner *et al*, 1989). More recently, tin-17m-DTPA (Atkins *et al*, 1995) rhenium-188-HEDP (Palmedo *et al*, 2000) and rhenium-188-DMSA (Blower *et al*, 2000) have been reported to be effective for bone pain palliation.

We have had more than 5 years experience in the use of ^188^Re-HEDP for the palliative treatment of bone metastases. Rhenium-188-HEDP has a rapid renal excretion (40±12% of administered activity within the first 8 h), leading to a low radiation dose to the whole body (0.07±0.02 mGy MBq^−1^) and a relative short biological half-life (51±43 h) in comparison to the half-life within bone metastases (269±166 h) (Liepe *et al*, 2003). The favourable physical characteristics of the radionuclide for its use in palliative therapy are a short physical half-life of 16.9 h and a maximal *β*-energy of 2.1 MeV with a 15% *γ*-component of 155 keV. This *γ*-component allows the control of tissue distribution after therapy. As a product from in-house ^188^W/^188^Re generator, similar to a technetium generator, ^188^Re-HEDP show a vantage availability. The long useful shelf-life of the generator leads to lower costs in comparison to other radiopharmaceuticals like strontium-89, rhenium-186-HEDP and samarium-153-EDTMP.

## MATERIALS AND METHODS

### Preparation of ^188^Re-HEDP

Rhenium-188-HEDP was prepared as previously described by Lin *et al* (1997) and Palmedo *et al* (2000). ^188^Re-perrhenate was obtained from a 38 GBq alumina-based ^188^W/^188^Re generator (Knapp *et al*, 1997) (Oak Ridge National Laboratory, Oak Ridge, USA). The generator was eluted with 20–25 ml of 0.9% saline. The generator eluates were concentrated to about 1.2 ml using a tandem cation/anion concentration system (Guhlke *et al*, 2000), which consists of an Ag Plus cartridge (Alltech Associates, Deerfield, IL, USA) attached to a three-way stopcock connected at the outlet to the QMA anion trapping column SepPak® (Waters Corporation, Milford, MA, USA) anion-exchange column. The concentration system was housed in a Lucite shield.

In total, 8.3 mg HEDP (Fluka Chemie AG, Buchs, Switzerland), 3.0 mg gentisic acid (Sigma-Aldrich, Steinheim, Germany) and 3.9 mg stannous chloride dihydrate (Merck, Darmstadt, Germany) were weighed in kit vials and mixed with 1.0 ml of carrier-added ^188^Re-generator eluate (10 *μ*l ReO4 Aldrich, 100 *μ*mol ml^−1^ physiological saline). The solution was heated at 96–100°C for 15 min. After cooling to room temperature, 1 ml of a sterile 0.3 M sodium hydroxide solution was added to adjust the pH to a range of 5–6.

Quality control of carrier-added ^188^Re-HEDP was performed with thin layer chromatography using silica gel (ITLC-SG) strips (Gelman, Ann Arbor, MI, USA). In addition, anion exchange chromatography based on gradient elution with increasing concentrations of NaCl solutions using a QMA SepPak® was used. The radiochemical purity determined by both procedures (ITLC and ion exchange) was more than 90%. Sterility and pyrogenicity tests were performed for each preparation.

### Patients

**Figure 1:**
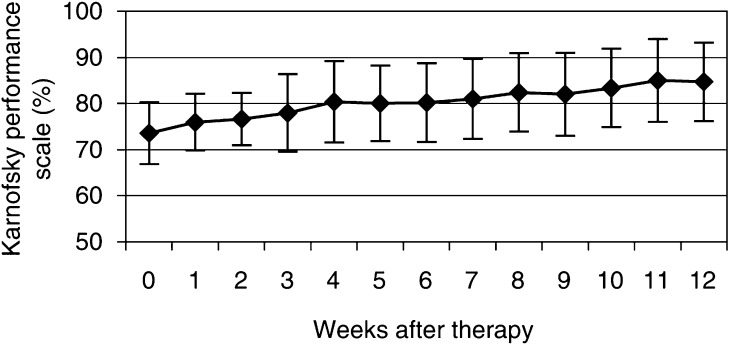
Time course of the Karnofsky performance scale before and within 12 weeks after ^188^Re-HEDP therapy (with s.d.'s).

In this phase II study, 27 patients with multiple bone metastases from prostate cancer and pain well-controlled by conventional analgesia were included. In previous dose-escalation studies (Palmedo *et al*, 2000), an activity of 3300 MBq of ^188^Re-HEDP was determined as suitable for bone pain palliation. We evaluated the effect of ^188^Re-HEDP on pain relief, reduction of analgesics and changes in quality of life. Before and for 12 weeks after the ^188^Re-HEDP therapy, weekly interviews with a standardised set of questions concerning pain relief with the visual analogue scale (VAS) from 0 to 100% (Serafini *et al*, 1998; Kraeber-Bodere *et al*, 2000), analgesics and Karnofsky performance scale were conducted weekly (Figure 1

). A pain relief ⩽25% on the VAS at least in two consecutive weeks without increase of analgesics was used as criteria for response.

Blood counts were obtained to evaluate the impairment of bone marrow function within 6 weeks after therapy and in the 12th week. For the assessment of toxicity, the toxicity criteria of the World Health Organization (1979) were used. According to this toxicity scale, platelet counts of 74–99*10^9^ l^−1^, 50–74*10^9^ l^−1^, 25–49*10^9^ l^−1^ and below 25*10^9^ l^−1^ correspond to a toxicity grade of 1, 2, 3 and 4, respectively. The maximum decrease was calculated by comparison of the level of blood counts before therapy (baseline) with the lowest level within 12 weeks. All patients underwent a ^99m^Tc-HMDP bone scan within 1 month before and 3 months after therapy to obtain a possible decrease of the mass of bone metastases. In a single case, we had also a later bone scan.

Criteria for patient inclusion in the study were

- positive ^99m^Tc-HMDP bone scan with at least three lesions;
- bone pain symptoms requiring the long-term use of analgesics;
- sufficient bone marrow function with a platelet count ⩾100 × 10^3^ *μ*l^−1^, leucocyte count ⩾3.0*10^3^ *μ*l^−1^ and haemoglobin ⩾6.0 mmol l^−1^ (9.67 g dl^−1^);
- normal renal function.

Criteria for exclusion were

- metastatic bone fractures;
- spinal cord compression;
- soft tissue tumours elsewhere causing nerve compression.

Chemotherapy and bisphosphonate therapy were discontinued 4 weeks before the administration of the radiopharmaceutical. In accordance with the Helsinki declaration, all patients were informed comprehensively about the study and possible side effects and were provided with a leaflet. Consent approval had been obtained from the local ethics committee.

### Statistics

Data are presented as mean±s.d. For determining a significant change in the visual analogue scale and Karnofsky performance scale, we used the Student's *t*-test. Two-tailed *P*-values less than 0.05 were considered to indicate statistical significance.

## RESULTS

Twenty-seven patients (mean age=68, range 55–87 years) with disseminated bone metastases from prostate cancer received a single intravenous injection of ^188^Re-HEDP. The administered activity ranged from 2700 to 3587 MBq (mean dose=3245±356 MBq). All patients had analgesics. Patients were hospitalised for 2 days for the ^188^Re-HEDP therapy due to regulations in the German radiation protection law. All patients had a single therapy. All patients received a hormone therapy for at least 6 months before therapy and also during the post-therapy observation period. Nineteen patients had been treated with a bisphosphonate for at least 6 months, this was discontinued four weeks before therapy. In addition, five patients received chemotherapy and seven patients external beam radiation prior to the ^188^Re-HEDP therapy. All these treatments were finished at least 5 months before the ^188^Re-HEDP therapy. It is unlikely that these various preceding therapies modified or altered the observed therapeutic effect in this study.

Twenty-five patients were included in the evaluation of the therapeutic efficiency of the ^188^Re-HEDP therapy. Two patients were excluded: one patient because of incomplete follow-up and one died 9 weeks after treatment from the cancer, unrelated to the therapy.

**Figure 2:**
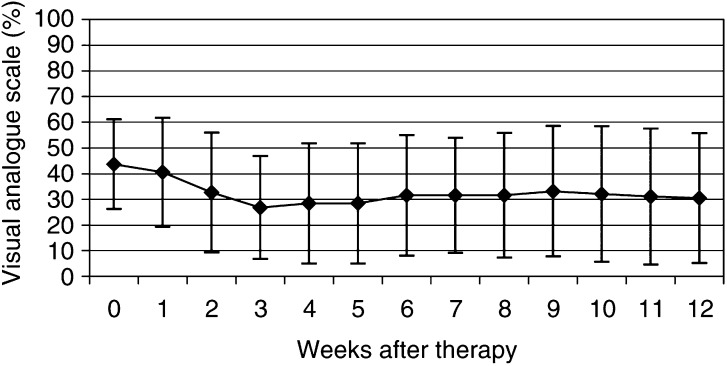
Time course of the pain at visual analogue scale before and within 12 weeks after ^188^Re-HEDP therapy (with s.d.'s).

The Karnofsky performance scale increased from 74±7% before therapy to 85±9% at 12 weeks after therapy (Figure 1). This increase was statistically significant (*P*=0.001). Pain relief ⩽25% on the VAS at least in two consecutive weeks without increase of analgesics intake was achieved in 76% of patients (19 out of 25). Five from these 19 patients were pain free (20%). The VAS showed a decrease from 44±18 to 31±25% at the 12th week after ^188^Re-HEDP therapy (*P*=0.009) (Figure 2

). The maximum of pain relief was described within the 3rd to the 8th week (27±20%).

**Figure 3:**
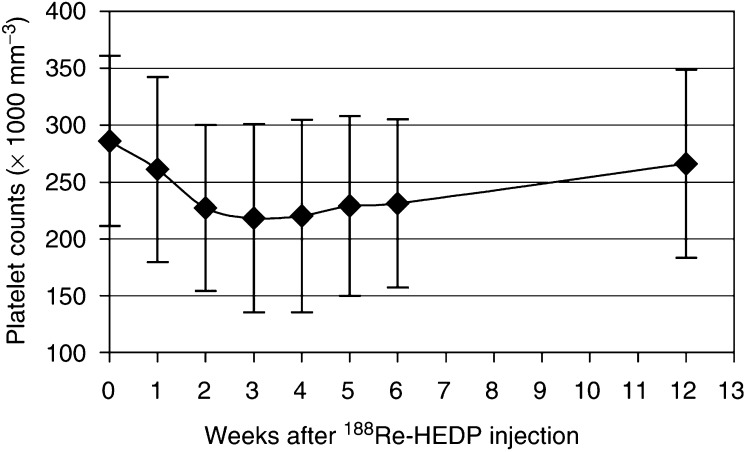
Bone marrow impairment due to ^188^Re-HEDP expressed as platelet counts in serial blood samples (with s.d.'s).

**Figure 4:**
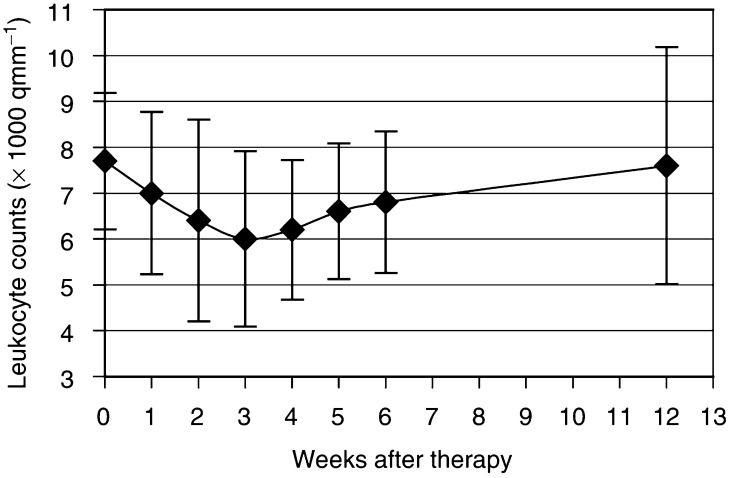
Bone marrow impairment due to ^188^Re-HEDP expressed as leucocyte counts in serial blood samples (with s.d.'s).

There was no evidence of either local or systemic intolerance to treatment with ^188^Re-HEDP, while a flare reaction with an increase of pain within 14 days after therapy was noted in 16% of patients. Most of the patients showed a grade I anaemia according to the WHO criteria (World Health Organization, 1979) before injection, with no significant worsening within 12 weeks after therapy (haemoglobin 7.6±2.6 before and 7.1±3.0 mmol l^−1^ 12 weeks after treatment). The maximum decrease in one patient from the baseline value was 6.8 to 5.7 mmol l^−1^. There was mild thrombocytopaenia (grade I thrombocytopaenia in two patients and grade II in one patient), but no evidence of unacceptable toxicity (defined as grade III or IV toxicity). Thrombocytopaenia was reversible within 12 weeks after therapy. The platelet counts decreased from a baseline value of 286±75*10^9^ l^−1^ to a maximum of 218±83*10^9^ l^−1^ at 2.7±0.9 weeks after therapy (Figure 3

). The patient with a thrombocytopaenia grade II had a platelet count of 62*10^9^ l^−1^. Grade I leucopenia was observed only in one patient (2.9*10^9^ l^−1^) and was also reversible within 12 weeks. The leucocyte counts decreased from a baseline value of 7.4±1.5*10^9^ l^−1^–6.0±1.9*10^9^ l^−1^ at 3.0±0.6 weeks after therapy (Figure 4

).

Generally, over the follow-up period, there were no significant changes in the PSA levels, but in individual cases a decrease was noted (PSA level: 159±151 *vs* 155±203 ng ml^−1^; *P*=0.96).

**Figure 5:**
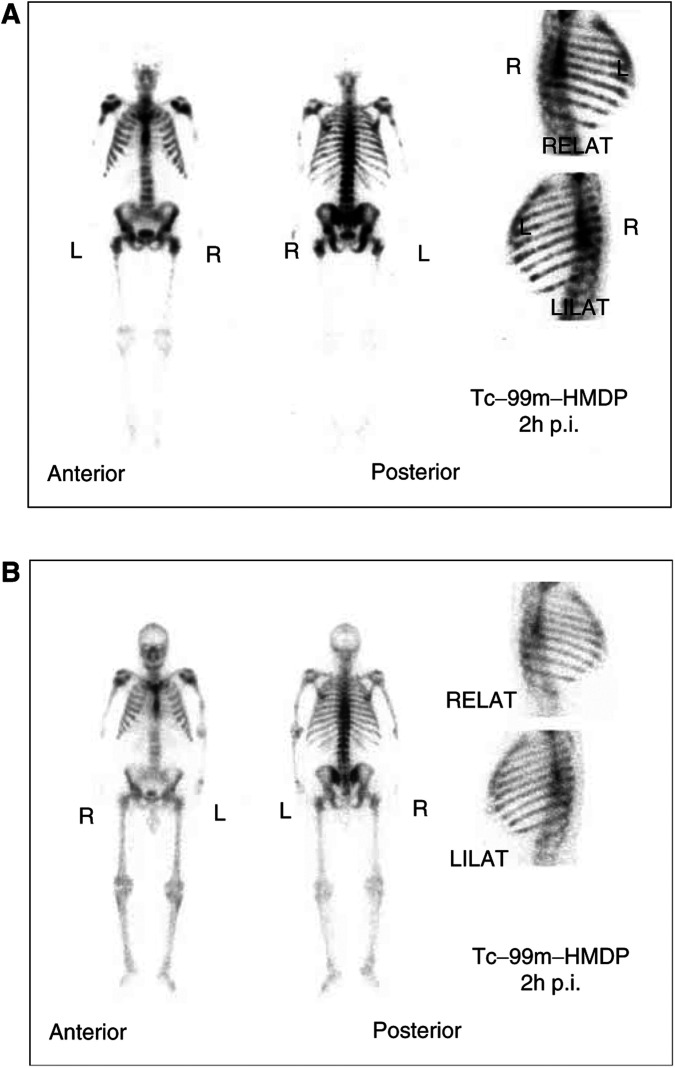
(**A**) Bone scan of a 71-year-old patient with bone metastases induced by prostate cancer one month before ^188^Re-HEDP therapy (bone scan index=45). The Karnofsky performance scale was 70% and the pain of the visual analogue scale=50%. (**B**) Bone scan of the same patient 6 months after therapy (bone scan index=17). The Karnofsky performance scale was 80% and the pain of the visual analogue scale=25%.

No patient showed a decrease in the bone metastases demonstrated on the ^99m^Tc-HMDP bone scans before treatment when compared to the scans obtained 3 months post-treatment. The bone scan index (BSI) level (Blake *et al*, 1986) in the group of patients with no response to the ^188^Re-HEDP therapy was 54±19, in the group of patients with a good response 37±14 and in pain-free patients 36±5 (*P*=0.24). In three of the patients, there was a decrease of the number of bone metastases with a mean decrease of the BSI level from 33±18 before treatment to 19±11 within 1 year after ^188^Re-HEDP therapy. One of these showed an improvement of the BSI from 45 to 17 six months after therapy (Figure 5A, B

). The 71-year-old patient was treated by bisphosphonate therapy (with etidronacid), nonsteroid antiandrogen (flutimide), progestagen (megestrolacetate) and an analogue of the luteinising hormone (goserelineacetate) from the beginning of the disease. Under this therapy, the patient described a progression of the disease with an increase in pain symptoms. Within 12 weeks of the ^188^Re-HEDP therapy, the Karnofsky performance scale increased from 70 to 80%, and the pain at the VAS decreased from 50 to 25%.

## DISCUSSION

Systemic radionuclide therapy with *β*-emitting radiopharmaceuticals represents a therapeutic option in the management of intractable metastatic bone pain and has been used since 1942 (Pecher, 1942). Nowadays, strontium-89, rhenium-186-HEDP and samarium-153-EDTMP are the preferred radiopharmaceuticals. From a theoretical point of view, ^188^Re-HEDP offers potential as a new and attractive radiopharmaceutical for the treatment. As a generator product it has an excellent availability that permits on-site labelling as with the routinely used ^99m^Tc-generator resulting in low costs.

In the literature there are few reports about the therapeutic efficiency of ^188^Re-HEDP in only small patient groups (Maxon *et al*, 1998; Palmedo *et al*, 2000), which reported a response rate of 70–80%. In 23 patients with bone metastases from a variety of primary tumours, ^188^Re-HEDP therapy was able to reduce analgesic intake in 82% of patients (Chen, 2000). In addition, 70% of the patients had a significant and 22% a minor improvement of quality of life. Using ^188^Re-HEDP at an activity of 1100 MBq, a response rate of 80% was obtained in a cohort of 61 patients with various primary tumours (Li *et al*, 2001). Studies with a large number of patients exist for patients treated with strontium-89, rhenium-186-HEDP and samarium-153-EDTMP. In a multicentre study with 818 patients treated with strontium-89 and rhenium-186-HEDP Dafermou *et al* (2001) described pain relief in 81% of the patients with prostate cancer and 27% of these were pain free. Enrique *et al* (2002)showed a response rate of 73% in a cohort of 417 patients treated with samarium-153-EDTMP at an activity varying between 18.5 and 55 MBq kg^−1^ body weight. In our study using ^188^Re-HEDP, pain relief was demonstrated in 76% of patients and 20% from these were pain free without increase of analgesia. In addition, a significant increase of the Karnofsky performance scale of 11% compared to the initial value within 12 weeks was observed. In comparison, the dose-escalation study in a small number of patients (*n*=6) using 3300 MBq of ^188^Re-HEDP (Palmedo *et al*, 2000) showed a lower response rate.

In the present study, no unacceptable toxicity was observed. There were two patients with thrombocytopaenia grade I and one with thrombocytopaenia grade II. A mean decrease of platelet counts of 30±14% from the baseline value was noticed, maximally at 2.7±0.9 weeks after therapy. The maximum decrease in one patient was down to 62*10^9^ l^−1^. The leucocyte counts showed a mean decrease of 25±17% from the baseline value (Figure 4) with a maximum at 3.0±0.6 weeks. Only one patient had a leucopenia grade I. The bone marrow toxicity of ^188^Re-HEDP showed no correlation to the extension of bone metastases in the ^99m^Tc-HMDP bone scan or the level of the BSI. Similar results are shown in the dose-escalation study with 3300 MBq ^188^Re-HEDP of Palmedo *et al* (2000), who observed two patients with thrombocytopaenia grade I and one with grade II. In the group with 4400 MBq, an unacceptable toxicity was observed in three out of eight patients, two with thrombocytopaenia grade IV and one with thrombocytopaenia grade III. Therefore, an activity of 3300 MBq of ^188^Re-HEDP is generally accepted in the palliative treatment of bone metastases.

The mean PSA level was unchanged during the follow-up (159±151 *vs* 155±203 ng ml^−1^; *P*=0.96, but in individual cases there was a measurable decrease in the PSA.

While radionuclide therapy for bone pain is mainly palliative, occasionally cure can be obtained and in the three cases described in this study there were decreases in bone metastases shown on conventional ^99m^Tc-HMDP bone scan with concomitant decrease in the BSI level from 33±18 to 19±11 within 1 year after treatment (Robinson *et al*, 1989; Mertens *et al*, 1992; Liepe *et al*, 2000). Of these patients, two were pain free.

In general, large bone metastases demonstrated on the ^99m^Tc-HMDP bone scans correlate well with sites of pain. In patients where there was no response to therapy, a higher BSI level was seen (BSI=51±19) compared to those patients where a favourable response occurred or the patients were pain free (BSI=33±11) (*P*=0.08). Other authors also described a higher response rate to treatment in early bone metastases, favouring active treatment in such cases (Kraeber-Bodere *et al*, 2000; Dafermou *et al*, 2001).

A previous dosimetry study gave the radiation absorbed dose for ^188^Re-HEDP as 11.8±6.2 Gy (range: 1.2–31.3 Gy) for the bone metastases, and 1.9±0.7 Gy (1.3–3.6 Gy) for the red bone marrow (Liepe *et al*, 2003). These values are comparable with the dosimetry for ^186^Re-HEDP (26 Gy for the bone metastases and 1.8 Gy for the red bone marrow) and also with the therapeutic effect and the bone marrow toxicity of ^186^Re-HEDP (Maxon *et al*, 1990,^1998^).

In conclusion, ^188^Re-HEDP is effective in the palliation of metastatic bone pain, without induction of severe side effects.
